# Safety and tolerability of monthly galcanezumab injections in patients with migraine: integrated results from migraine clinical studies

**DOI:** 10.1186/s12883-020-1609-7

**Published:** 2020-01-17

**Authors:** Mark E. Bangs, David Kudrow, Shufang Wang, Tina M. Oakes, Gisela M. Terwindt, Delphine Magis, Laura Yunes-Medina, Virginia L. Stauffer

**Affiliations:** 1 Eli Lilly and Company, Lilly Corporate Center, Indianapolis, IN 46285 USA; 2 California Medical Clinic for Headache, Santa Monica, CA USA; 3 Leiden University Medical Center, Leiden, The Netherlands; 4 Neurology Dept and Pain Clinic (CMTD), CHR East Belgium, Verviers, Belgium

**Keywords:** CGRP, Galcanezumab, Migraine, Safety, Tolerability

## Abstract

**Background:**

Galcanezumab, a humanized monoclonal antibody that selectively binds to calcitonin gene-related peptide, has demonstrated a significant reduction in monthly migraine headache days in phase 2 and 3 trials. In these analyses, we aimed to evaluate the safety and tolerability of galcanezumab compared with placebo for prevention of episodic or chronic migraine.

**Methods:**

Data were integrated from three double-blind clinical studies for the up to 6-month galcanezumab exposure group (*N* = 1435), and from five clinical studies for the up to 1-year all-galcanezumab exposure group (*N* = 2276). Patients received a monthly 120 mg subcutaneous injection of galcanezumab (with a 240 mg loading dose in month 1), 240 mg galcanezumab, or placebo. Outcomes measured were treatment-emergent adverse events (TEAEs), serious AEs (SAEs), and discontinuation due to AEs (DCAEs). Laboratory results, vital signs, electrocardiogram (ECG), suicidal ideation and behavior results were evaluated.

**Results:**

TEAEs that occurred more frequently in galcanezumab-treated patients included injection site pain, injection site reactions excluding pain, constipation, vertigo, and pruritus. The proportion of DCAEs among galcanezumab-treated patients ranged between 1.8 and 3.0%, and differed from placebo group for galcanezumab 240 mg (*P* < 0.05). Fewer than 2.0% of patients in either galcanezumab dose-group compared with 1.0% of placebo-treated patients reported a SAE. There were no clinically meaningful differences between galcanezumab and placebo in laboratory measures, vital signs including blood pressure, ECGs, cardiovascular-related AEs, or suicidal ideation and behavior.

**Conclusions:**

Galcanezumab demonstrated a favorable safety and tolerability profile for up to 1 year of treatment for the prevention of migraine.

**Trial registration:**

Clinical Trials CGAB = NCT02163993, EVOLVE-1 = NCT02614183, EVOLVE-2 = NCT02614196, REGAIN = NCT02614261, and CGAJ = NCT02614287. All were first posted on 25 November 2015, except CGAB posted on 16 June 2014, and before enrolling the first patient.

## Background

Calcitonin gene-related peptide (CGRP), found in a variety of anatomical locations, has a diverse role, and evidence of its presence during migraine attacks has been established [1–4]. It facilitates the production and secretion of pro-inflammatory mediators, and its role in the pathophysiology of pain is widely recognized [5, 6].

CGRP is a potent microvascular vasodilator with various physiological roles [7]; therefore, specific concerns exist regarding a potential CGRP antagonism associated with hemodynamic or other cardiovascular (CV)-related adverse events (AEs) [8]. CV comorbidities, such as hypertension, and risk of ischemic CV events like stroke, myocardial infarction and heart disease are increased in the migraine population compared with nonmigraine populations [9–14]. The relationship between migraine and CV disease is still not fully understood and needs further attention; underlying pathophysiological mechanisms may play a role such as increased vascular vulnerability [8].

Galcanezumab is a humanized monoclonal antibody that is approved for the preventive treatment of migraine and the treatment of episodic cluster headache to reduce the frequency of migraine headaches and cluster headache attacks; it potently and selectively binds CGRP preventing its biological activity without blocking the CGRP receptor [15]. Phase 2 and 3 migraine prevention studies showed that galcanezumab reduced monthly migraine headache days and had a favorable short-term safety and tolerability compared with placebo [16–24].

A recent meta-analysis of randomized controlled trials of CGRP monoclonal antibodies for the prevention of episodic migraine supported the findings of safety and tolerability of CGRP monoclonal antibodies [25]. The meta-analysis included ten phase 2 and phase 3 clinical trials; four of those trials were galcanezumab studies. The studies included had a duration of up to 6 months, which prompted the authors to question the long-term safety and tolerability of CGRP monoclonal antibodies.

Our objective here is to report integrated safety data compared with placebo for up to 6 months in a large cohort of patients with migraine and to confirm the safety and tolerability profile in patients receiving galcanezumab up to 1 year.

## Methods

### Study design

Data from three double-blind phase 3 studies (EVOLVE-1 [19], EVOLVE-2 [20], and REGAIN [21]), were integrated and included once-monthly, subcutaneous doses of galcanezumab (120 mg [with an initial loading dose of 240 mg] and 240 mg) compared with placebo (Table 1). Integration of data was justified since all studies were placebo controlled, all used the same randomization ratios of placebo: galcanezumab 120 mg: galcanezumab 240 mg dose-groups (2:1:1), and had comparable study designs. Furthermore, the safety results among the individual studies were consistent.

**Table 1:** Overview of all clinical studies

Study	Trial registration	Design	Population	Treatment period	Randomization ratios	Randomized patients		Patient completion
CGAB^a^	NCT02163993	Phase 2b, randomized, DB, placebo-controlled dose-ranging study	Patients with episodic migraine	3-month DB treatment phase	2:1:1:1:1	*N* = 410		Completed DB Treatment Phase: 375 Completed Post-treatment (washout): 361
						PBO	137	
						GMB 5 mg	68	
						GMB 50 mg	68	
						GMB 120 mg	70	
						GMB 300 mg	67	
EVOLVE-1	NCT02614183	Phase 3, randomized, DB, placebo-controlled, parallel group study	Patients with episodic migraine	6-month DB treatment phase	2:1:1	*N* = 858		Completed DB Treatment Phase: 703 Completed Post-treatment (washout): 704
						PBO	433	
						GMB 120 mg	213	
						GMB 240 mg	212	
EVOLVE-2	NCT02614196	Phase 3, randomized, DB, placebo-controlled, parallel group study	Patients with episodic migraine	6-month DB treatment phase	2:1:1	*N* = 915		Completed DB Treatment Phase: 785 Completed Post-treatment (washout): 797
						PBO	461	
						GMB 120 mg	231	
						GMB 240 mg	223	
REGAIN^b^	NCT02614261	Phase 3, randomized, DB, placebo-controlled, parallel group study	Patients with chronic migraine	3-month DB treatment phase; 9-month OL treatment phase	2:1:1	*N* = 1113		Completed DB Treatment Phase: 1037 Completed OL Treatment: 555 Completed Post-treatment (washout): 218
						PBO	558	
						GMB 120 mg	278	
						GMB 240 mg	277	
CGAJ	NCT02614287	Phase 3, randomized, long-term, OL safety study	Patients with episodic or chronic migraine	12-month OL treatment phase	1:1	*N* = 270		Completed OL Treatment: 210 Completed Post-treatment (washout): 198
						GMB 120 mg	135	
						GMB 240 mg	135	

Abbreviations: *DB* Double-blind, *GMB* Galcanezumab, *NCT* clinicaltrials.gov registration, *OL* Open-label, *PBO* Placebo

^a^Only the patients in the GMB 120 mg dose-group were included in the analyses

^b^Given the flexible dosing of the open-label treatment phase, some patients had a modal treatment that is different from their randomized treatment. A patient who was randomized to GMB 120 mg in the DB treatment phase and then received additional injections of GMB 240 mg in the flexible OL treatment phase could eventually have a modal dose of GMB 240 mg

The data for all-galcanezumab exposures from patients treated with 120 mg or 240 mg of galcanezumab in phase 2 and 3 migraine prevention studies which included the 9-month open-label extension phase for REGAIN, the 1-year safety study CGAJ [22], and the 3-month double-blind study CGAB [18] are also presented (Table 1). The all-galcanezumab exposure group is used to compare longer exposure time to galcanezumab to the integrated double-blind studies that exposed patients to galcanezumab for a shorter period. Trial registration information is presented in Table 1.

### Participants

The inclusion and exclusion criteria for all studies have been published previously [17–21]. Key exclusion criteria included presence of a medical condition that would preclude study participation including pregnancy, suicidal ideation within the past month, history of substance abuse or dependence in the past year, lifetime history of stroke (REGAIN and EVOLVE-2) or within 6 months of screening (CGAB, EVOLVE-1 and CGAJ), and patients at-risk for acute (within 6 months of screening) or serious CV events as judged by the investigator. Patients with other comorbid CV conditions were included.

Patients were categorized into the CV disease risk subgroup “yes” if the patient reported one or more pre-existing or medical history events included in the narrow search terms of the following standard Medical Dictionary for Regulatory Activities (MedDRA® v.19.1) queries (SMQs): Ischemic heart disease, Hypertension, Cardiac failure, Cardiomyopathy, Ischemic central nervous system vascular conditions, Dyslipidemia, and Hyperglycemia/new onset diabetes mellitus; patients who did not report any of these conditions prior to study randomization were categorized as “no” for CV disease risk group.

### Procedures

Each of the studies included objectives to compare the safety and tolerability of galcanezumab with placebo in patients with episodic or chronic migraine using the following measures: treatment-emergent adverse events (TEAEs), serious adverse events (SAEs), discontinuation due to adverse events (DCAE), laboratory measures, temperature, blood pressure (BP), pulse, weight, suicidal ideation/behavior, and electrocardiogram (ECG).

### Outcomes

In these studies, an adverse drug reaction (ADR) was identified by the study sponsor as a clinical event reasonably associated with galcanezumab treatment. Using medical judgement, all AEs and numerical safety data were evaluated for a possible causal relationship to galcanezumab exposure. Factors used to determine the list of ADRs included the following: a statistical assessment of the effect via odds ratios and significance, any dose relationship, biologic plausibility, clinical relevance of any individual case (e.g., any available de-challenge/re-challenge information), the severity of the event, the consistency of findings across studies, similar events, and similar compounds.

The assessment of hypersensitivity events was conducted using the Hypersensitivity SMQ. Medical review of each case identified by the SMQ was conducted to determine whether the identified terms represented events that were likely hypersensitivity in nature.

Changes from baseline for continuous laboratory analyses were assessed and included a complete blood cell panel, clinical chemistry, and urinalysis.

Changes in hepatic function were assessed by treatment-emergent (TE) changes in hepatic laboratory measures and defined as any change from a baseline normal (i.e., ≤ 1 times the upper limit of normal [ULN]) to a post-baseline abnormal high. Those tests included alanine aminotransferase or aspartate aminotransferase (either ≥3, 5, or 10 times the ULN); alkaline phosphatase (≥ 2 times ULN), or total bilirubin (≥ 2 times the ULN).

At every office visit, temperature was collected, BP and pulse were measured in triplicate (values were averaged for each visit and recorded as such) in the sitting position prior to blood draws and administration of study drug, and suicidal ideation and behavior was assessed using the Columbia-Suicide Severity Rating Scale (C-SSRS). The C-SSRS scale captures the occurrence, severity, and frequency of suicide-related thoughts and behaviors during the assessment period [26]. A single, 12-lead digital ECG was collected at baseline and endpoint in the double-blind studies. Weight was collected at baseline and endpoint.

### Statistical analysis

Time-at-risk-adjusted incidence rates per 100 patient-years, as well as unadjusted proportions, were calculated for TEAEs, SAEs, and DCAEs except for AEs related to injection sites, as these events mainly occur on the day of injection, a time-at-risk adjustment is not appropriate due to non-constant hazard over time. Treatment comparisons for proportions for categorical data (TEAE, SAE, DCAE, and TE abnormal hepatic laboratory measures, TE ECG findings, vital signs, and C-SSRS) were analyzed using the Cochran–Mantel–Haenszel test stratified by study. Treatment comparisons for time-at-risk-adjusted incidence rates were from likelihood ratio test from a Poisson regression with study and treatment as independent variables and time at risk as offset term. Treatment comparisons in mean change of continuous measures (laboratory measures, quantitative ECG measures, and vital signs) were analyzed using analysis of covariance models containing terms of treatment, study, and baseline measurement (Type III sums of squares).

All analyses were conducted for the safety population (all patients who were randomized and treated with at least one dose of study drug), and results were summarized by patient’s modal treatment group during study period of interest. Statistical comparisons (*p*-values) were conducted as exploratory without control for multiple testing. The statistical package used was SAS Enterprise Guide 7.0 (SAS Institute, Cary, NC).

## Results

### Patients demographics and disposition

#### Integrated double-blind phase 3 studies

The safety population (*N* = 2886) was an average age of 41.0 years, mostly female (84.5%), white (76.7%), and from North America (67.4%). Baseline characteristics by treatment group are summarized in Table 2. Based on reported medical history obtained prior to study initiation, 17.2 to 18.5% of all patients were identified in the CV disease risk-subgroup designation “yes”. The most common conditions in the CV disease risk group were related to hypertension (placebo = 10.3%, galcanezumab = 7.9%), dyslipidemia (placebo = 9.0%, galcanezumab = 10.2%), and hyperglycemia/new onset diabetes mellitus (placebo = 3.2%, galcanezumab = 2.9%). Patients in these three studies reflected the general population of patients with migraine with regard to demographics, disease characteristics, and comorbid medical conditions [27]. For the REGAIN study, the protocol allowed for up to one third of patients to continue on a stable dose of either topiramate or propranolol for migraine prevention, but only 14.6% of patients randomized were receiving a concomitant medication for migraine prevention; most of those patients received topiramate (10.3%) compared to propranolol (4.3%). Additionally, among patients with chronic migraine in the REGAIN study, 64% met the criteria for medication overuse and medication overuse headache was reported at baseline at similar rates among the treatment groups (placebo = 63.4%, galcanezumab 120 mg = 64.3%, and galcanezumab 240 mg = 64.1%).

**Table 2:** Baseline patient demographics and disease characteristics of the integrated double-blind studies

		Galcanezumab	
	Placebo *N* = 1451 n (%)	120 mg *N* = 705 n (%)	240 mg *N* = 730 n (%)
Age			
< 30 years	269 (18.5)	139 (19.7)	158 (21.6)
≥ 30 and < 40 years	342 (23.6)	182 (25.8)	194 (26.6)
≥ 40 and < 50 years	435 (30.0)	199 (28.2)	192 (26.3)
≥ 50 years	405 (27.9)	185 (26.2)	186 (25.5)
Sex			
Male	214 (14.8)	106 (15.0)	121 (16.6)
Female	1237 (85.3)	599 (85.0)	609 (83.4)
Race			
American Indian or Alaska Native	24 (1.7)	10 (1.4)	16 (2.2)
Asian	89 (6.1)	48 (6.8)	42 (5.8)
Black or African American	117 (8.1)	53 (7.5)	59 (8.1)
Native Hawaiian, Other Pacific Islander	2 (0.1)	0 (0.0)	4 (0.6)
White	1112 (76.6)	545 (77.3)	555 (76.1)
Multiple	107 (7.4)	49 (7.0)	53 (7.3)
Ethnicity			
Hispanic or Latino	298 (20.5)	149 (21.1)	170 (23.3)
Not Hispanic or Latino	1077 (74.2)	522 (74.0)	530 (72.6)
Region			
North America	977 (67.3)	472 (67.0)	496 (68.0)
Europe	262 (18.1)	126 (17.9)	131 (18.0)
Other	212 (14.6)	107 (15.2)	103 (14.1)
Comorbid conditions occurring in > 10% of any dose-group			
Seasonal allergy	307 (21.2)	158 (22.4)	122 (16.7)a
Drug hypersensitivity	247 (17.0)	123 (17.5)	128 (17.5)
Insomnia	165 (11.4)	89 (12.6)	78 (10.7)
Anxiety	166 (11.4)	82 (11.6)	81 (11.1)
Depression	181 (12.5)	84 (11.9)	79 (10.8)
Back pain	151 (10.4)	62 (8.8)	75 (10.3)
Cardiovascular disease risk group^b^			
Yes	269 (18.5)	123 (17.5)	124 (17.0)
No	1182 (81.5)	582 (82.6)	606 (83.0)

Abbreviations: *MedDRA* Medical Dictionary for Regulatory Activities, *N* Number of patients in the analysis population with non-missing demographic measures, *n* Number of patients within each specific category

^a^Indicates a statistically significant difference (*P* < 0.05) compared with placebo

^b^The following were standardized MedDRA® (certified terminology, and the international medical terminology developed under the auspices of the International Council for Harmonisation of Technical Requirements for Pharmaceuticals for Human Use) queries used to identify patients in the “yes” subgroup if patients affirmed one or more of the following as pre-existing or in their medical history: Ischaemic heart disease (including myocardial infarction, and other ischaemic heart disease), Hypertension, Cardiac failure, Cardiomyopathy, Ischaemic central nervous system vascular conditions, Dyslipidaemia and Hyperglycaemia/new onset diabetes mellitus

Among all three studies, 89.1% of patients treated with galcanezumab completed double-blind treatment compared with 85.9% of placebo-treated patients. The most frequently reported reasons for discontinuation were patient decision, lost to follow-up, and AEs. No deaths were reported in any of the three studies. A total of 1435 patients were exposed to galcanezumab during the placebo-controlled phase of the three studies, which represented 536.3 patient-years of exposure (galcanezumab 120 mg = 267.7 patient-years and galcanezumab 240 mg = 268.7 patient-years).

#### All-galcanezumab exposure

The demographics of this population were similar when compared to the integrated double-blind studies population. Across the five studies, a total of 2276 patients were exposed to galcanezumab 120 mg or galcanezumab 240 mg, representing 1416.5 patient-years of exposure. A total of 526 patients were exposed to galcanezumab for a year. Table 1 presents patient disposition for all studies.

### Adverse events

An overview of AEs by treatment group is shown in Table 3. The percentages and exposure adjusted incidence rates (EAIR) were higher for the galcanezumab-treated patients in the integrated double-blind studies compared to placebo. However, the EAIR did not increase with longer duration as shown in the all-galcanezumab exposure group. TEAEs that occurred in at least 1.0% of patients in either galcanezumab dose-group and more frequently than in the placebo dose-group during double-blind treatment are presented in Table 4. Injection site pain, injection site reactions excluding injection site pain, constipation, pruritus (not associated with injection site), and vertigo were considered ADRs associated with galcanezumab treatment.

**Table 3:** Overview of adverse events

	Integrated double-blind studies									All galcanezumab exposures					
	Placebo N = 1451			GMB 120 mg N = 705			GMB 240 mg N = 730			GMB 120 mg *N* = 926			GMB 240 mg *N* = 1350		
	%	n/TPY	EAIR (95% CI)	%	n/TPY	EAIR (95% CI)	%	n/TPY	EAIR (95% CI)	%	n/TPY	EAIR (95% CI)	%	n/TPY	EAIR (95% CI)
Deaths		0			0			0			0			0	
SAEs^a^	1.0	14/530.37	2.64 (1.44,4.43)	1.7	12/264.47	4.54 (2.34,7.93)	1.5	11/266.95	4.12 (2.06,7.37)	2.8	26/521.05	4.99 (3.26,7.31)	3.0	40/876.06	4.57 (3.26,6.22)
DCAEs	1.7	24/530.08	4.53 (2.90,6.74)	1.8	13/266.26	4.88 (2.60,8.35)	3.0	22/266.50	8.26 (5.17,12.50)	4.0	37/524.43	7.06 (4.97,9.72)	3.9	52/882.74	5.89 (4.40,7.72)
TEAEs^a^	57	827/295.25	280.10 (261.34,299.86)	62.6	441/145.16	303.80 (276.10,333.52)	64.7	472/133.62	353.25 (322.10,386.61)	68.8	664/228.62	290.44 (268.77,313.40)	74.4	897/308.13	291.11 (272.37,310.80)

Abbreviations: *CI* Confidence interval, *DCAEs* Adverse events leading to discontinuation, *EAIR* Time-at-risk incidence rate, *GMB* Galcanezumab, *N* Number of patients in the analysis population, *n* Number of patients in specific category, *SAEs* Serious adverse events, *TEAEs* Treatment-emergent adverse events, *TPY* Total patient-year-at-risk

^a^ In ≥1 patient

**Table 4:** Treatment-emergent adverse events with a ≥ 1.0% frequency of occurrence in either galcanezumab dose-group

	Integrated double-blind studies						All galcanezumab exposures			
	Placebo *N* = 1451		GMB 120 mg *N* = 705		GMB 240 mg *N* = 730		GMB 120 mg *N* = 991		GMB 240 mg *N* = 1285	
	%	EAIR (95% CI)	%	EAIR (95% CI)	%	EAIR (95% CI)	%	EAIR (95% CI)	%	EAIR (95% CI)
Injection site pain^a^	9.5		10.1		11.6		11.0		9.6	
Nasopharyngitis^b^	6.5	18.19 (14.70,22.26)	7.4	20.13 (15.04,26.40)	4.3^c^	11.85 (8.05,16.82)	9.4	20.13 (16.25,24.66)	8.5	16.55 (13.59,19.97)
Upper respiratory tract infection	4.1	11.52 (8.79,14.83)	4.4	11.88 (8.07,16.86)	4.9	13.70 (9.60,18.97)	6.8	14.30 (11.08,18.16)	7.0	13.47 (10.83,16.56)
Injection site reaction^a^	1.0		3.1^d^		6.2^d^		5.2		6.5	
Dizziness	2.8	7.85 (5.63,10.65)	2.8	7.65 (4.67,11.82)	2.7	7.61 (4.65,11.75)	2.8	5.90 (3.92,8.53)	3.3	6.24 (4.50,8.44)
Injection site erythema^a^	1.4		2.8^c^		4.0^d^		3.2		4.4	
Sinusitis	2.1	5.88 (3.99,8.34)	2.8	7.59 (4.64,11.73)	2.6	7.18 (4.32,11.21)	4.1	8.62 (6.18,11.69)	3.9	7.39 (5.48,9.74)
Urinary tract infection	2.3	6.29 (4.33,8.83)	2.7	7.20 (4.33,11.24)	2.5	6.78 (4.02,10.72)	2.6	5.42 (3.54,7.94)	3.8	7.23 (5.35,9.56)
Fatigue	2.3	6.49 (4.50,9.07)	2.4	6.49 (3.78,10.39)	2.2	6.07 (3.47,9.86)	2.2	4.61 (2.89,6.98)	2.7	5.03 (3.48,7.03)
Injection site pruritus^a^	0.1		2.1^d^		3.3^d^		2.3		2.8	
Neck pain	1.5	3.97 (2.46,6.07)	2.1	5.65 (3.16,9.32)	0.8	2.25 (0.82,4.89)	1.7	3.53 (2.06,5.66)	1.7	3.22 (2.02,4.87)
Abdominal pain	1.7	4.56 (2.92,6.79)	1.8	4.92 (2.62,8.41)	0.8	2.24 (0.82,4.88)	1.9	3.95 (2.38,6.17)	1.3	2.48 (1.44,3.97)
Cough	1.3	3.59 (2.16,5.61)	1.7	4.53 (2.34,7.90)	1.8	4.88 (2.60,8.35)	1.8	3.74 (2.22,5.92)	2.5	4.68 (3.20,6.61)
Oropharyngeal pain	0.9	2.46 (1.31,4.20)	1.4	3.77 (1.81,6.94)	1.6	4.51 (2.33,7.87)	1.9	3.95 (2.38,6.17)	2.1	3.95 (2.61,5.75)
Bronchitis	1.2	3.21 (1.87,5.15)	1.3	3.38 (1.55,6.42)	1.5	4.13 (2.06,7.39)	1.8	3.72 (2.21,5.88)	2.7	4.98 (3.45,6.95)
Influenza	2.3	6.46 (4.47,9.02)	1.1	3.01 (1.30,5.93)	2.7	7.55 (4.61,11.66)	2.3	4.79 (3.04,7.19)	4.1	7.69 (5.74,10.08)
Constipation	0.6	1.51 (0.65,2.97)	1.0	2.64 (1.06,5.44)	1.5^c^	4.14 (2.07,7.40)	1.3	2.70 (1.44,4.61)	1.3	2.48 (1.44,3.96)
Migraine	1.0	2.64 (1.44,4.43)	1.0	2.62 (1.05,5.41)	1.6	4.50 (2.32,7.86)	0.8	1.65 (0.71,3.25)	1.7	3.21 (2.01,4.86)
Rash	1.2	3.22 (1.87,5.15)	1.1	3.02 (1.30,5.94)	1.4	3.76 (1.80,6.91)	1.8	3.73 (2.21,5.90)	2.0	3.65 (2.36,5.39)
Weight increased	0.8	2.27 (1.17,3.96)	1.3	3.38 (1.55,6.42)	0.8	2.24 (0.82,4.88)	1.9	3.95 (2.38,6.17)	1.4	2.62 (1.55,4.15)
Influenza-like illness	0.6	1.51 (0.65,2.97)	0.9	2.25 (0.83,4.90)	1.1	3.00 (1.29,5.90)	0.9	1.86 (0.85,3.53)	1.6	2.92 (1.78,4.51)
Injection site bruising^a^	0.6		0.6		1.4		1.2		1.6	
Pruritus (not related to injection site)	0.3	0.75 (0.20,1.93)	0.7	1.88 (0.61,4.38)	1.2^c^	3.37 (1.54,6.40)	1.4	2.90 (1.59,4.87)	1.3	2.47 (1.44,3.95)
Vertigo	0.2	0.56 (0.12,1.65)	0.7	1.88 (0.61,4.38)	1.2^c^	3.39 (1.55,6.43)	1.0	2.07 (0.99,3.80)	1.1	2.04 (1.11,3.42)
Dyspepsia	0.6	1.70 (0.78,3.22)	0.7	1.88 (0.61,4.38)	1.1	3.00 (1.30,5.92)	0.9	1.86 (0.85,3.52)	1.1	2.03 (1.11,3.41)
Hypertension	1.0	2.83 (1.58,4.67)	1.1	3.01 (1.30,5.93)	0.7	1.87 (0.61,4.36)	1.1	2.27 (1.14,4.07)	0.9	1.74 (0.90,3.05)
Anxiety	0.9	2.45 (1.31,4.19)	1.3	3.39 (1.55,6.44)	0.4	1.12 (0.23,3.27)	1.7	3.53 (2.06,5.65)	0.9	1.74 (0.90,3.04)
Injection site swelling^a^	0.1		1.1^d^		0.6^c^		1.2		0.8	
Viral infection	0.8	2.07 (1.04,3.71)	1.1	3.01 (1.30,5.93)	0.6	1.49 (0.41,3.82)	1.1	2.27 (1.13,4.06)	0.5	1.01 (0.41,2.09)
Nasal congestion	0.6	1.51 (0.65,2.97)	0.4	1.12 (0.23,3.28)	1.1	3.00 (1.30,5.91)	0.6	1.24 (0.45,2.69)	0.9	1.60 (0.80,2.85)

Abbreviations: *CI* Confidence interval, *EAIR* Exposure-adjusted incidence rate, *GMB* Galcanezumab, *N* Number of patients in the analysis population; n, number of patients in specific category

^a^Common adverse events related to injection sites are not included in EAIR analyses as they do not satisfy the constant hazard assumption of EAIR calculation

^b^Events occurred in the galcanezumab 240 mg dose group less frequently than placebo

^c^*P* < 0.05 compared with placebo

^d^*P* < 0.001 compared with placebo

### Injection site related adverse events

Injection site pain was the most frequently reported of these events. The incidence was similar among treatment groups in the integrated double-blind studies and the all-galcanezumab exposure group (Table 4). Longer exposure to galcanezumab did not increase the frequency of injection site pain reported by galcanezumab-treated patients. Among patients in the integrated double-blind studies who reported injection site pain, regardless of the treatment group, most reported that the events occurred within 60 min of the injection (84.9%), were mild to moderate in severity, occurred on the day of injection, and resolved in less than 3 days (galcanezumab 120 mg = 2.8 days, galcanezumab 240 mg = 2.7 days, and placebo = 2.0 days). Galcanezumab-treated patients in the integrated double-blind studies who reported AEs related to injection sites other than injection site pain (galcanezumab 120 mg = 9.9%, *P* < 0.001; galcanezumab 240 mg = 14.5%, *P* < 0.001) did so at a higher rate than those treated with placebo (4.1%).

No SAEs related to injection sites were reported. Seven patients discontinued galcanezumab treatment due to injection site-related AEs (Table 9).

### Treatment-emergent adverse events

Constipation, pruritus (not associated with injection site), and vertigo were reported at a higher frequency in the galcanezumab treated patients compared with placebo (Table 4). The galcanezumab 240 mg dose-group was statistically different compared to placebo for these TEAEs (*P* < 0.05). Most of the constipation and vertigo TEAEs were mild or moderate in severity, while the pruritus TEAEs were mild in severity. No patient discontinued due to these TEAEs. The EAIR of these TEAEs in the all-galcanezumab exposure 120 mg and 240 mg dose-group did not increase with longer treatment compared to integrated double-blind studies. In the patients with chronic migraine, there were no statistically significant differences in common TEAEs (≥1.5% for galcanezumab treatment groups and greater than placebo) reported by patients with or without concurrent prophylaxis use and patients with or without medication overuse (Table 5 and Table 6).

**Table 5:** Common treatment-emergent adverse events by concurrent prophylaxis use in patients with chronic migraine

	Concurrent prophylaxis use			No concurrent prophylaxis use			Interaction *p*-value^a^
	Placebo (*N* = 82)	GMB 120 mg (*N* = 37)	GMB 240 mg (*N* = 43)	Placebo (*N* = 476)	GMB 120 mg (*N* = 236)	GMB 240 mg (*N* = 239)	
	n (%)	n (%)	n (%)	n (%)	n (%)	n (%)	
Patients with ≥ 1 TEAE	42 (51.22)	21 (56.76)	22 (51.16)	237 (49.79)	138 (58.47)	138 (57.74)	0.751
Common TEAE							
Injection site pain	2 (2.44)	4 (10.81)	1 (2.33)	22 (4.62)	13 (5.51)	19 (7.95)	0.162
Nasopharyngitis	4 (4.88)	3 (8.11)	1 (2.33)	22 (4.62)	14 (5.93)	8 (3.35)	0.875
Injection site reaction	1 (1.22)	0 (0.00)	1 (2.33)	9 (1.89)	8 (3.39)	14 (5.86)	0.839
Upper respiratory tract infection	2 (2.44)	1 (2.70)	1 (2.33)	11 (2.31)	8 (3.39)	8 (3.35)	0.970
Back pain	1 (1.22)	1 (2.70)	0 (0.00)	13 (2.73)	8 (3.39)	2 (0.84)	0.887
Fatigue	1 (1.22)	1 (2.70)	0 (0.00)	9 (1.89)	5 (2.12)	6 (2.51)	0.689
Injection site erythema	1 (1.22)	1 (2.70)	2 (4.65)	4 (0.84)	3 (1.27)	11 (4.60)	0.777
Abdominal pain	2 (2.44)	2 (5.41)	0 (0.00)	7 (1.47)	4 (1.69)	4 (1.67)	0.629
Diarrhea	2 (2.44)	1 (2.70)	1 (2.33)	7 (1.47)	2 (0.85)	5 (2.09)	0.776
Sinusitis	1 (1.22)	0 (0.00)	2 (4.65)	4 (0.84)	4 (1.69)	6 (2.51)	0.800
Urinary tract infection	2 (2.44)	0 (0.00)	2 (4.65)	5 (1.05)	6 (2.54)	2 (0.84)	0.249
Neck pain	1 (1.22)	1 (2.70)	0 (0.00)	7 (1.47)	6 (2.54)	0 (0.00)	0.773
Migraine	1 (1.22)	0 (0.00)	1 (2.33)	4 (0.84)	5 (2.12)	3 (1.26)	0.718
Influenza like illness	2 (2.44)	1 (2.70)	0 (0.00)	1 (0.21)	4 (1.69)	4 (1.67)	0.274
Arthralgia	0 (0.00)	0 (0.00)	2 (4.65)	5 (1.05)	1 (0.42)	3 (1.26)	0.471
Oropharyngeal pain	0 (0.00)	0 (0.00)	1 (2.33)	3 (0.63)	2 (0.85)	4 (1.67)	0.898
Injection site pruritus	0 (0.00)	0 (0.00)	0 (0.00)	1 (0.21)	0 (0.00)	7 (2.93)	0.473
Pyrexia	0 (0.00)	1 (2.70)	0 (0.00)	2 (0.42)	4 (1.69)	1 (0.42)	0.932

Abbreviations: *GMB* Galcanezumab, *N* Number of subjects in safety population, *n* Number of subjects within each specific category, *TEAE* Treatment-emergent adverse events

^a^*P*-values: TEAE indicator (Y/N) = treatment, baseline medication overuse, concurrent prophylaxis use, and treatment by subgroup

**Table 6:** Common treatment-emergent adverse events by medication overuse in patients with chronic migraine

	Medication overuse			No medication overuse			Interaction *p*-value^a^
	Placebo (*N* = 353)	GMB 120 mg (*N* = 178)	GMB 240 mg (*N* = 177)	Placebo (*N* = 204)	GMB 120 mg (*N* = 94)	GMB 240 mg (*N* = 104)	
	n (%)	n (%)	n (%)	n (%)	n (%)	n (%)	
Patients with ≥ 1 TEAE	169 (47.88)	99 (55.62)	99 (55.93)	110 (53.92)	59 (62.77)	60 (57.69)	0.800
Common TEAE							
Injection site pain	15 (4.25)	9 (5.06)	11 (6.21)	9 (4.41)	8 (8.51)	9 (8.65)	0.734
Nasopharyngitis	13 (3.68)	11 (6.18)	6 (3.39)	13 (6.37)	6 (6.38)	3 (2.88)	0.597
Injection site reaction	10 (2.83)	5 (2.81)	12 (6.78)	0 (0.00)	3 (3.19)	3 (2.88)	0.209
Upper respiratory tract infection	10 (2.83)	6 (3.37)	6 (3.39)	3 (1.47)	3 (3.19)	3 (2.88)	0.795
Back pain	9 (2.55)	6 (3.37)	2 (1.13)	5 (2.45)	3 (3.19)	0 (0.00)	0.789
Fatigue	5 (1.42)	3 (1.69)	4 (2.26)	5 (2.45)	3 (3.19)	2 (1.92)	0.771
Injection site erythema	3 (0.85)	1 (0.56)	6 (3.39)	2 (0.98)	3 (3.19)	7 (6.73)	0.596
Abdominal pain	7 (1.98)	3 (1.69)	1 (0.56)	2 (0.98)	3 (3.19)	2 (1.92)	0.340
Diarrhea	7 (1.98)	2 (1.12)	3 (1.69)	2 (0.98)	1 (1.06)	3 (2.88)	0.566
Sinusitis	2 (0.57)	2 (1.12)	7 (3.95)	3 (1.47)	2 (2.13)	1 (0.96)	0.216
Urinary tract infection	3 (0.85)	4 (2.25)	2 (1.13)	4 (1.96)	2 (2.13)	1 (0.96)	0.727
Neck pain	5 (1.42)	4 (2.25)	0 (0.00)	3 (1.47)	3 (3.19)	0 (0.00)	0.946
Migraine	4 (1.13)	3 (1.69)	3 (1.69)	1 (0.49)	2 (2.13)	1 (0.96)	0.771
Influenza like illness	2 (0.57)	3 (1.69)	2 (1.13)	1 (0.49)	2 (2.13)	2 (1.92)	0.929
Arthralgia	4 (1.13)	0 (0.00)	2 (1.13)	1 (0.49)	1 (1.06)	3 (2.88)	0.351
Oropharyngeal pain	2 (0.57)	1 (0.56)	3 (1.69)	1 (0.49)	1 (1.06)	2 (1.92)	0.922
Injection site pruritus	0 (0.00)	0 (0.00)	4 (2.26)	1 (0.49)	0 (0.00)	3 (2.88)	0.729
Pyrexia	1 (0.28)	2 (1.12)	0 (0.00)	1 (0.49)	3 (3.19)	1 (0.96)	0.856

Abbreviations: *GMB* Galcanezumab, *N* Number of subjects in safety population, *n* Number of subjects within each specific category, *TEAE* Treatment-emergent adverse events

^a^*P*-values: TEAE indicator (Y/N) = treatment, baseline medication overuse, concurrent prophylaxis use, and treatment by subgroup Note: 3 patients in the analysis population had missing medication overuse

### Hypersensitivity

As shown in Table 7, there was a higher frequency of patients in the galcanezumab 120 mg dose-group and galcanezumab 240 mg dose-group reporting a hypersensitivity-related AE compared with placebo. No reports of anaphylaxis occurred. In the galcanezumab-treated patients, the severity of these events were 4.8% mild, 1.1% moderate, and 0.2% severe.

**Table 7:** Percentage of patients in integrated double-blind studies who experienced a hypersensitivity event^a,b^

		Galcanezumab	
	Placebo *N* = 1451 n (%)	120 mg *N* = 705 n (%)	240 mg *N* = 730 n (%)
Hypersensitivity SMQ	40 (2.8)	36 (5.1)^c^	33 (4.5)
Rash	17 (1.2)	8 (1.3)	10 (1.4)
Injection site rash	2 (0.1)	6 (0.9)^c^	4 (0.6)
Dermatitis contact	4 (0.3)	3 (0.4)	4 (0.6)
Rhinitis allergic	3 (0.2)	3 (0.4)	4 (0.6)
Hypersensitivity	0 (0.0)	3 (0.4)^c^	2 (0.3)^c^
Injection site hypersensitivity	0 (0.0)	1 (0.1)	3 (0.4)^c^
Dermatitis allergic	0 (0.0)	3 (0.4)^c^	0 (0.0)
Eczema	3 (0.2)	1 (0.1)	2 (0.3)
Rash pruritic	0 (0.0)	2 (0.3)^c^	1 (0.1)
Urticaria	5 (0.3)	2 (0.3)	1 (0.1)
Injection site urticaria	1 (0.1)	1 (0.1)	1 (0.1)
Rash generalized	1 (0.1)	1 (0.1)	1 (0.1)
Rash papulosquamous	0 (0.0)	1 (0.1)	1(0.1)
Dermatitis atopic	1 (0.1)	0 (0.0)	1(0.1)
Eye allergy	0 (0.0)	1 (0.1)	0 (0.0)
Pruritus allergic	0 (0.0)	1 (0.1)	0 (0.0)
Rash erythematous	1 (0.1)	0 (0.0)	1 (0.1)
Rash maculo-papular	1 (0.1)	1 (0.1)	0 (0.0)
Bronchospasm	1 (0.1)	0 (0.0)	0 (0.0)
Conjunctivitis allergic	1 (0.1)	0 (0.0)	0 (0.0)
Drug hypersensitivity	1 (0.1)	0 (0.0)	0 (0.0)

Abbreviations: *MedDRA* Medical Dictionary for Regulatory Activities, *N* Number of patients in the analysis population, *n* Number of patients within each specific category; SMQ, standardized MedDRA query

^a^These are likely events per medical review

^b^Categorized by narrow standardized MedDRA®^b^ (v.19.1) query search terms. MedDRA®, the certified Medical Dictionary for Regulatory Activities terminology, is the international medical terminology developed under the auspices of the International Council for Harmonisation of Technical Requirements for Pharmaceuticals for Human Use

^c^Indicates a statistically significant difference (*P* < 0.05) compared with placebo

Urticaria was a hypersensitivity event that occurred in less than 0.3% of all-galcanezumab- and placebo-treated patients. Two patients reported an SAE of non-immediate urticaria (occurred days after the last injection) during the open-label treatment period of the REGAIN study; neither patient reported other associated symptoms. Owing to the biological plausibility of the association between galcanezumab and these two SAEs, urticaria has been designated as an ADR associated with galcanezumab treatment. Neither of these two patients were positive for TE anti-drug antibodies.

### Serious adverse events

Fewer than 2.0% of patients in either galcanezumab dose-group reported SAEs (Table 8); the percentage among placebo-treated patients was 1.0%. A review of all SAEs did not reveal any patterns or notable differences between galcanezumab- and placebo-treated patients, nor between galcanezumab dose-groups (Table 8). Furthermore, the EAIRs for SAEs reported by at least one patient demonstrated no trend of an increase with longer treatment duration (Table 3).

**Table 8:** Serious adverse events by system organ class that occurred in either galcanezumab dose-groups compared with placebo during double-blind treatment

		Galcanezumab	
	Placebo *N* = 1451 n (%)	120 mg *N* = 705 n (%)	240 mg *N* = 730 n (%)
Patients with at least one SAE	14 (1.0)	12 (1.7)	11 (1.5)
Cardiac disorders	1 (0.1)	0 (0.0)	1 (0.1)
Acute myocardial infarction	0 (0.0)	0 (0.0)	1 (0.1)
Myocardial infarction	1 (0.7)	0 (0.0)	0 (0.0)
Gastrointestinal disorders	3 (0.2)	4 (0.6)	1 (0.1)
Pancreatitis acute	0 (0.0)	1 (0.1)	1 (0.1)
Gastritis	1 (0.1)	1 (0.1)	0 (0.0)
Rectal polyp	0 (0.0)	1 (0.1)	0 (0.0)
Small intestinal obstruction	0 (0.0)	1 (0.1)	0 (0.0)
Alcoholic pancreatitis	1 (0.1)	0 (0.0)	0 (0.0)
Hemorrhoids	1 (0.1)	0 (0.0)	0 (0.0)
General disorders and administration site conditions	0 (0.0)	0 (0.0)	1 (0.1)
Pyrexia	0 (0.0)	0 (0.0)	1 (0.1)
Hepatobiliary disorders	3 (0.2)	0 (0.0)	1 (0.1)
Cholelithiasis	2 (0.1)	0 (0.0)	1 (0.1)
Gallbladder polyp	1 (0.1)	0 (0.0)	0 (0.0)
Infections and infestations	0 (0.0)	1 (0.1)	1 (0.1)
Influenza	0 (0.0)	0 (0.0)	1 (0.1)
Injury, poisoning and procedural complications	1 (0.1)	2 (0.3)	1 (0.1)
Incarcerated incisional hernia	0 (0.0)	1 (0.1)	0 (0.0)
Ligament rupture	0 (0.0)	1 (0.1)	0 (0.0)
Meniscus injury	0 (0.0)	0 (0.0)	1 (0.1)
Seroma	0 (0.0)	1 (0.1)	0 (0.0)
Foot fracture	1 (0.1)	0 (0.0)	0 (0.0)
Rib fracture	1 (0.1)	0 (0.0)	0 (0.0)
Road traffic accident	1 (0.1)	0 (0.0)	0 (0.0)
Metabolism and nutrition disorders	0 (0.0)	0 (0.0)	1 (0.1)
Hypokalemia	0 (0.0)	0 (0.0)	1 (0.1)
Musculoskeletal and connective tissue disorders	1 (0.1)	1 (0.1)	0 (0.0)
Tendonitis	0 (0.0)	1 (0.1)	0 (0.0)
Vertebral osteophyte	1 (0.1)	0 (0.0)	0 (0.0)
Neoplasms benign, malignant and unspecified	0 (0.0)	3 (0.4)	0 (0.0)
Adenocarcinoma of the cervix	0 (0.0)	1 (0.2)	0 (0.0)
Colon cancer	0 (0.0)	1 (0.1)	0 (0.0)
Tubular breast carcinoma	0 (0.0)	1 (0.1)	0 (0.0)
Nervous system disorders	1 (0.1)	0 (0.0)	2 (0.3)
Generalized tonic-clonic seizure	0 (0.0)	0 (0.0)	1 (0.1)
Transient ischemic attack	0 (0.0)	0 (0.0)	1 (0.1)
Migraine	1 (0.1)	0 (0.0)	0 (0.0)
Psychiatric disorders	1 (0.1)	0 (0.0)	1 (0.1)
Disorientation	0 (0.0)	0 (0.0)	1 (0.1)
Suicide attempt	1 (0.1)	0 (0.0)	0 (0.0)
Renal and urinary disorders	0 (0.0)	1 (0.1)	2 (0.3)
Bladder dysfunction	0 (0.0)	1 (0.1)	0 (0.0)
Nephrolithiasis	0 (0.0)	0 (0.0)	1 (0.1)
Renal colic	0 (0.0)	0 (0.0)	1 (0.1)
Respiratory, thoracic and mediastinal disorders	2 (0.1)	0 (0.0)	1 (0.1)
Pulmonary embolism	1 (0.1)	0 (0.0)	1 (0.1)
Epistaxis	1 (0.1)	0 (0.0)	0 (0.0)
Vascular disorders	1 (0.1)	0 (0.0)	0 (0.0)
Deep vein thrombosis	1 (0.1)	0 (0.0)	0 (0.0)

Abbreviations: *N* Number of patients in the analysis population, *n* Number of patients within each specific category, *SAE* Serious adverse event

There were no SAEs reported more than once with the exception of acute pancreatitis (*n* = 2), which was reported by one patient in each of the two galcanezumab dose-groups; cholelithiasis was reported by two placebo-treated patients. Regarding the SAEs of pancreatitis, one was in a patient in the galcanezumab 240 mg dose-group. Five days after the first dose of galcanezumab the patient was admitted to the hospital with epigastric pain which had started prior to treatment randomization; lipase was elevated (14,663 units per litre [normal range is 73–393 units per litre]). Ultrasonography showed significant sludge in the gallbladder, and the computed tomography scan was consistent with pancreatitis of the pancreatic tail. The patient was discharged from the hospital, re-admitted twice in the following weeks, and finally underwent an endoscopic sphincterotomy and laparoscopic cholecystectomy, which showed numerous pigmented black gallbladder stones. A likely cause for pancreatitis in this patient was the presence of the gallbladder stones; additionally, this event was confounded by concomitant topiramate use. The patient discontinued from the study. In the opinion of the investigator, the pancreatitis was not related to study drug treatment. The other SAE of pancreatitis was reported by a patient in the galcanezumab 120 mg dose-group. Four days after the second dose of galcanezumab, the patient presented with abdominal pain and was diagnosed with pancreatitis; lipase level was only slightly above the ULN (411 units per litre). The abdominal computed tomography report stated that the patient had ileus of the distal small bowel which could explain the abdominal symptoms, but was not consistent with pancreatitis. A similar incident occurred in this patient previously. The event resolved, and the patient completed both the treatment and post-treatment periods of the study. Again, in the opinion of the investigator, pancreatitis was not related to the treatment with galcanezumab.

### Cardiovascular related serious adverse events

CV-related SAEs were reported at a similar frequency by galcanezumab- and placebo-treated patients, and included myocardial infarction, pulmonary embolism, deep vein thrombosis, and transient ischemic attack (Table 8).

### Discontinuations due to adverse events

Although the percentage of DCAEs was not more than 3.0% among all treatment groups, DCAEs were statistically different among galcanezumab 240 mg treated patients compared to placebo (Table 9). AE leading to discontinuation in at least two galcanezumab-treated patients were as follows: worsening of migraine (*n* = 5), injection site reaction (*n* = 4), hepatic enzyme increased (*n* = 2), nasopharyngitis (n = 2), and weight increase (n = 2). The EAIRs for DCAEs demonstrated no trend of an increase with longer treatment duration (Table 3).

**Table 9:** Adverse events leading to discontinuation of treatment by system organ class that occurred in either galcanezumab dose-groups compared with placebo during double-blind treatment

		Galcanezumab	
	Placebo *N* = 1451 n (%)	120 mg *N* = 705 n (%)	240 mg *N* = 730 n (%)
Patients with DCAE	24 (1.7)	13 (1.8)	22 (3.0)^a^
Cardiac disorder	1 (0.1)	0 (0.0)	0 (0.0)
Myocardial infarction	1 (0.1)	0 (0.0)	0 (0.0)
Ear and labyrinth disorder	1 (0.1)	0 (0.0)	0 (0.0)
Vertigo	1 (0.1)	0 (0.0)	0 (0.0)
Gastrointestinal disorders	1 (0.1)	1 (0.1)	2 (0.3)
Dyspepsia	0 (0.0)	0 (0.0)	1 (0.1)
Gastritis	0 (0.0)	1 (0.1)	0 (0.0)
Pancreatitis acute	0 (0.0)	0 (0.0)	1 (0.1)
Abdominal pain	1 (0.1)	0 (0.0)	0 (0.0)
General disorders and administration site conditions	2 (0.1)	3 (0.4)	7 (1.0)
Injection site reaction	0 (0.0)	1 (0.1)	3 (0.4)^†^
Chest discomfort	0 (0.0)	0 (0.0)	1 (0.1)
Influenza-like illness	0 (0.0)	0 (0.0)	1 (0.1)
Injection site erythema	0 (0.0)	1 (0.1)	0 (0.0)
Injection site pain	0 (0.0)	0 (0.0)	1 (0.1)
Injection site swelling	0 (0.0)	0 (0.0)	1 (0.1)
Peripheral swelling	0 (0.0)	1 (0.1)	0 (0.0)
Facial pain	1 (0.1)	0 (0.0)	0 (0.0)
Fatigue	1 (0.1)	0 (0.0)	0 (0.0)
Hepatobiliary disorders	1 (0.1)	0 (0.0)	0 (0.0)
Cholelithiasis	1 (0.1)	0 (0.0)	0 (0.0)
Immune system disorders	0 (0.0)	0 (0.0)	1 (0.1)
Hypersensitivity	0 (0.0)	0 (0.0)	1 (0.1)
Infections and infestations	1 (0.1)	0 (0.0)	3 (0.4)
Nasopharyngitis	0 (0.0)	0 (0.0)	2 (0.3)^†^
Infection	0 (0.0)	0 (0.0)	1 (0.1)
Tinea capitis	1 (0.1)	0 (0.0)	0 (0.0)
Investigations	0 (0.0)	2 (0.3)	2 (0.3)
Hepatic enzyme increased	0 (0.0)	0 (0.0)	2 (0.3)^†^
Weight increase	0 (0.0)	2 (0.3)^†^	0 (0.0)
Musculoskeletal and connective tissue disorders	3 (0.2)	1 (0.1)	0 (0.0)
Myalgia	0 (0.0)	1 (0.1)	0 (0.0)
Arthralgia	1 (0.1)	0 (0.0)	0 (0.0)
Pain in extremity	1 (0.1)	0 (0.0)	0 (0.0)
Vertebral osteophyte	1 (0.1)	0 (0.0)	0 (0.0)
Neoplasms benign, malignant and unspecified	0 (0.0)	2 (0.3)	0 (0.0)
Adenocarcinoma of the cervix^b^	0 (0.0)	1 (0.2)	0 (0.0)
Tubular breast carcinoma	0 (0.0)	1 (0.1)	0 (0.0)
Nervous system disorders	7 (0.5)	1 (0.1)	4 (0.6)
Migraine	3 (0.2)	1 (0.1)	4 (0.6)
Dizziness	1 (0.1)	0 (0.0)	0 (0.0)
Headache	1 (0.1)	0 (0.0)	0 (0.0)
Sciatica	1 (0.1)	0 (0.0)	0 (0.0)
Syncope	1 (0.1)	0 (0.0)	0 (0.0)
Psychiatric disorders	3 (0.2)	0 (0.0)	1 (0.1)
Depression	1 (0.1)	0 (0.0)	1 (0.1)
Generalized anxiety disorder	1 (0.1)	0 (0.0)	0 (0.0)
Suicide attempt	1 (0.1)	0 (0.0)	0 (0.0)
Respiratory, thoracic and mediastinal disorders	0 (0.0)	1 (0.1)	1 (0.1)
Bronchiectasis	0 (0.0)	1 (0.1)	0 (0.0)
Dyspnoea	0 (0.0)	0 (0.0)	1 (0.1)
Skin and subcutaneous tissue disorders	2 (0.1)	2 (0.3)	1 (0.1)
Rash, generalized	0 (0.0)	1 (0.1)	0 (0.0)
Rash, pruritic	0 (0.0)	1 (0.1)	0 (0.0)
Skin ulcer	0 (0.0)	0 (0.0)	1 (0.1)
Alopecia	1 (0.1)	0 (0.0)	0 (0.0)
Dermatitis atopic	1 (0.1)	0 (0.0)	0 (0.0)
Vascular disorders	2 (0.1)	0 (0.0)	0 (0.0)
Deep vein thrombosis	1 (0.1)	0 (0.0)	0 (0.0)
Hypertension	1 (0.1)	0 (0.0)	0 (0.0)

Abbreviations: *DCAE* Discontinued treatment due to an adverse event, *N* Number of patients in the analysis population, *n* Number of patients within each specific category

^a^Indicates a statistically significant difference (*P* < 0.05) compared with placebo

^b^Denominator adjusted for female-specific event

### Discontinuations due to cardiovascular related adverse events

CV-related AEs leading to discontinuation in placebo-treated patients were myocardial infarction, deep vein thrombosis, and hypertension (Table 9). The specific CV-related AE leading to discontinuation in one galcanezumab-treated patient was transient ischemic attack.

### Laboratory assessments including hepatic laboratory measures

There were no significant or clinically meaningful differences in mean change from baseline in the analyses of laboratory parameters for patients in galcanezumab-treated group compared with placebo. Galcanezumab treatment was not associated with any significant or clinically meaningful abnormalities of chemistry, hematology, or urinalysis laboratory analytes; mean changes in routine laboratory parameters were generally minimal. For laboratory values related to glucose and lipid metabolism, no clinically significant mean changes were observed.

There were no clinically relevant differences between patients treated with galcanezumab or placebo in the incidence of TE hepatic laboratory changes, the frequencies for any of these abnormalities were less than 1.0% (Table 10). Overall, for patients with TE hepatic laboratory changes, there were no patterns that would suggest an association with galcanezumab. No Hy’s Law cases were observed in any patient during these studies.

**Table 10:** Percentage of patients in integrated double-blind studies with treatment-emergent high hepatic laboratory measures at any time^a^

Analyte	Placebo	Galcanezumab	
		120 mg	240 mg
	*N* = 1202 n (%)	*N* = 591 n (%)	*N* = 598 n (%)
ALT ≥3x ULN	4 (0.3)	1 (0.2)	2 (0.3)
ALT ≥5x ULN	0 (0.0)	1 (0.2)	1 (0.2)
ALT ≥10x ULN	0 (0.0)	1 (0.2)	0 (0.0)
	*N* = 1274 n (%)	*N* = 635 n (%)	*N* = 636 n (%)
AST ≥3x ULN	1 (0.1)	1 (0.2)	1 (0.2)
AST ≥5x ULN	0 (0.0)	0 (0.0)	0 (0.0)
AST ≥10x ULN	0 (0.0)	0 (0.0)	0 (0.0)
	*N* = 1295 n (%)	*N* = 648 n (%)	*N* = 658 n (%)
ALP ≥2x ULN	0 (0.0)	0 (0.0)	0 (0.0)
	*N* = 1306 n (%)	*N* = 647 n (%)	*N* = 660 n (%)
TBIL ≥2x ULN	0 (0.0)	0 (0.0)	0 (0.0)

Abbreviations: *ALT* Alanine aminotransferase, *ALP* Alkaline phosphatase, *AST* Aspartate aminotransferase, *N* Number of patients in the baseline category and at least one postbaseline measurement, *n* Number of patients with at least one value in the postbaseline category, *TBIL* Total bilirubin, *ULN* Upper limit of normal

^a^Includes all patients with at least one post-baseline measurement and those with missing baseline values

### Vital signs, weight, and ECGs

Mean changes in temperature were not statistically different among any treatment group in these studies.

During the double-blind treatment period, mean changes from baseline to endpoint in systolic and diastolic BP, and pulse were minimal (≤1.0 mmHg and ≤ 2 bpm, respectively). The frequency of TE or sustained increases in BP, and pulse at any time was similar in galcanezumab- and placebo-treated patients, with no statistically significant or clinically meaningful differences (Table 11). Exposure-adjusted analysis of TE high systolic BP showed that in the integrated double-blind studies the EAIR was 8.7 for the galcanezumab 120 mg dose-group and 9.6 for the galcanezumab 240 mg dose-group, which was similar to placebo (8.1, IRR = 1.1–1.2). The exposure-adjusted analysis of TE high systolic BP showed that in the all-galcanezumab exposure group the EAIRs decreased for both treatment groups, 5.3 for the galcanezumab 120 mg and 8.7 for the galcanezumab 240 mg dose-group, suggesting that the TE increases in systolic BP did not increase with longer treatment duration. The analysis for TE high diastolic BP resulted in an EAIR of 21.4 for the galcanezumab120 mg dose-group and 19.3 for the galcanezumab 240 mg dose-group which was similar to placebo (17.6, IRR = 1.1–1.2). The analysis for TE high diastolic BP in the all-galcanezumab exposure group resulted in an EAIR of 16.5 for the galcanezumab 120 mg dose-group and 16.9 for the galcanezumab 240 mg dose-group, demonstrating that the TE increases in diastolic BP did not increase with longer treatment duration.

**Table 11:** Percentage of patients in integrated double-blind studies reporting treatment-emergent changes to blood pressure and pulse

		Galcanezumab	
	Placebo n/N (%)	120 mg n/N (%)	240 mg n/N (%)
Systolic blood pressure^a^			
Low	7/1399 (0.5)	3/698 (0.4)	8/706 (1.1)
High	40/1332 (3.0)	22/672 (3.3)	24/674 (3.6)
PCS high	1/1409 (0.1)	1/704 (0.1)	0/708 (0.0)
Sustained	7/1297 (0.5)	2/658 (0.3)	1/657 (0.2)
Diastolic blood pressure^b^			
Low	6/1404 (0.4)	2/704 (0.3)	8/709 (1.1)
High	83/1292 (6.4)	51/653 (7.8)	46/652 (7.1)
PCS high	8/1402 (0.6)	4/703 (0.6)	5/704 (0.7)
Sustained	15/1260 (1.2)	9/639 (1.4)	6/637 (0.9)
Pulse^c^			
Low	5/1403 (0.4)	1/700 (0.1)	2/707 (0.3)
High	32/1398 (2.3)	13/702 (1.9)	15/705 (2.1)
Sustained	1/1361 (0.1)	0/688 (0.0)	1/687 (0.2)

Abbreviations: *bpm* Beats per minute, *N* Number of patients in the analysis population, *n* Number of patients within each specific category, *PCS* Possibly clinically significant

^a^For systolic blood pressure: treatment-emergent low ≤90 mmHg with ≥20 mmHg decrease; treatment-emergent high ≥140 mmHg with ≥20 mmHg increase; PCS high ≥180 mmHg with ≥20 mmHg increase; sustained elevation ≥140 mmHg with ≥20 mmHg increase for 2 consecutive office visits

^b^For diastolic blood pressure: treatment-emergent low ≤50 mmHg with ≥10 mmHg decrease; treatment-emergent high ≥90 mmHg with ≥10 mmHg increase; PCS high ≥105 mmHg with ≥15 mmHg increase; sustained elevation ≥90 mmHg with ≥10 mmHg increase for 2 consecutive office visits

^c^For pulse: treatment-emergent low < 50 bpm with ≥15 bpm decrease; treatment-emergent high > 100 bpm with ≥15 bpm increase; sustained elevation > 100 bpm with ≥15 bpm increase for 2 consecutive office visits

A similar proportion of galcanezumab- and placebo-treated patients either gained or lost at least 7.0% of their baseline weight (gained ≥7.0%: galcanezumab 120 mg = 6.3%, galcanezumab 240 mg = 6.4%, placebo = 5.2%; lost ≥7.0%: galcanezumab 120 mg = 3.9%, galcanezumab 240 mg = 3.4%, placebo = 2.6%), and there were no clinically important or statistically different changes in weight between any treatment-group in these studies.

For ECG analysis, there were no clinically meaningful differences observed between treatment groups. No patient met clinically significant increases in QTcF (> 500 msec).

### Suicidal ideation or behavior

Overall, data from galcanezumab-treated patients in these phase 3 studies do not suggest a risk of suicidal ideation or behavior as assessed by the C-SSRS. The integrated double-blind studies reported suicidal ideation in 13 (0.9%) patients in the placebo group, 4 (0.6%) patients in the galcanezumab 120 mg dose-group, and 7 (1.0%) patients in the galcanezumab 240 mg dose-group. Suicidal behavior was reported in 1 (0.1%) patient in the placebo group and in 1 (0.1%) patient in the galcanezumab 240 mg dose-group. The all-galcanezumab exposure group reported suicidal ideation in 11 (1.1%) patients in the galcanezumab 120 mg dose-group and 11 (0.9%) patients in the galcanezumab 240 mg dose-group. Suicidal behavior was reported in 1 (0.1%) patient in each galcanezumab dose-group.

## Discussion

In this integrated report of safety data from five clinical studies with up to a year of galcanezumab treatment, we have expanded available knowledge regarding the safety and tolerability of galcanezumab.

The most common AEs observed in our studies were those related to injection site, which are consistent with other marketed injectable CGRP monoclonal antibodies [28–36].

Although treatment with monoclonal antibodies can be associated with severe hypersensitivity adverse reactions, no anaphylaxis events were reported in these studies. However, two serious cases of non-immediate urticaria were reported in galcanezumab-treated patients during the open-label treatment phase of the REGAIN study. Hypersensitivity events generally occurred more frequently in patients treated with galcanezumab than with placebo, although most events were mild or moderate in severity and did not lead to discontinuation of galcanezumab treatment. A separate by-month analysis of hypersensitivity events (data on file) did not suggest a trend toward an increase in the frequency of hypersensitivity events with increased duration of treatment.

The events of constipation, vertigo, and pruritus were all reported in up to 1.0% of patients in the galcanezumab 120 mg dose-group and up to 1.5% of patients in the 240 mg dose-group. In the gastrointestinal system, CGRP affects visceral nociception, blood flow, inflammation, motility, colonic secretion, and is known to induce diarrhea in rodents [37], thus the ADR of constipation seems biologically plausible. Erenumab, another anti-CGRP medication, includes constipation as an ADR in product labeling [32]. Patients with migraine often report audiovestibular symptoms including vertigo, tinnitus, phonophobia, and hearing loss [38, 39]. Vertigo was categorized as an ADR since inhibition of CGRP may interfere with vestibular function [40, 41].

Differences between galcanezumab- and placebo-treated patients were observed in the percentage of SAEs and DCAEs; however, the types of events reported were similar and there was not a distinct pattern specific to galcanezumab treatment. There were low numbers of CV-related SAEs or DCAEs with a similar number of events reported in the placebo and galcanezumab treatment groups. All the CV-related SAEs in galcanezumab-treated patients resolved with no additional CV TEAEs or sequelae reported. In the patient with the transient ischemic attack, 2 computerized tomogram scans and a brain MRI (including angiography of the extracranial and intracranial vessels and ergometry) performed following the event were judged normal and without signs of ischemia. Additionally, treatment with galcanezumab or placebo did not lead to an increase in systolic or diastolic BP with up to a year of treatment.

Hepatic safety of galcanezumab was reported previously [16, 42]; no Hy’s Law observations were reported among any galcanezumab-treated patients or placebo in either of those previous reports, or in this report.

Although the pivotal galcanezumab studies included both the 120 mg (240 mg loading dose) and 240 mg doses administered monthly, the efficacy data in both the episodic migraine studies and the chronic migraine study consistently demonstrated the similarity across efficacy measures for both galcanezumab doses. Therefore, the approved dosage of galcanezumab is a monthly dose of 120 mg injected subcutaneously following an initial loading dose of 240 mg.

Galcanezumab offers the convenience and adherence benefits of once monthly dosing. In clinical practice, tolerability or unsatisfactory efficacy of current migraine preventive treatments often results in non-compliance and treatment failure [43, 44].

The span of the current clinical studies of galcanezumab has not been long enough to answer questions of long-term safety; however, no unexpected safety issues have emerged among these data that extend out to 1 year of treatment.

A meta-analysis of 10 randomized controlled trials of CGRP monoclonal antibodies for the prevention of episodic migraine was recently published [25]. This analysis supports the findings that CGRP monoclonal antibodies are generally safe and well tolerated. However, the meta-analysis did not include trials for the prevention of chronic migraine, which we have presented in this report for treatment with galcanezumab. In addition, other safety outcomes other than AEs were not included resulting in an incomplete presentation of the safety data in the meta-analysis. Finally, the meta-analysis only presented safety data for up to 6 months. In this report, we have presented safety and tolerability data for up to 1 year of exposure to galcanezumab and none of the safety outcomes assessed appear to increase in incidence with longer duration of treatment. Lastly there were no new AEs of concern that occurred with a treatment duration of up to 1 year.

A limitation of these integrated analyses is that rare AEs or long-term risks may not be evident in studies of shorter duration. Also, restrictions on the inclusion of patients with acute or serious CV risk or pregnant women may limit the generalizability of these results.

## Conclusion

In these studies, for up to 1 year of treatment, a favorable safety and tolerability profile for galcanezumab in the prevention of episodic and chronic migraine was evident. TEAEs, including those identified as ADRs due to their potential clinical significance, were generally transient in nature, resolved, and were amenable to monitoring. The proportion of DCAEs was low across all studies providing support for the tolerability of galcanezumab in patients with migraine. In addition, the overall safety profiles of galcanezumab 120 mg versus galcanezumab 240 mg in the integrated double-blind studies group and all-galcanezumab exposure group were similar.

## Data Availability

Lilly provides access to all individual participant data collected during the trial, after anonymization, with the exception of pharmacokinetic or genetic data. Data are available to request 6 months after the indication studied has been approved in the US and EU and after primary publication acceptance, whichever is later. No expiration date of data requests is currently set once data are made available. Access is provided after a proposal has been approved by an independent review committee identified for this purpose and after receipt of a signed data sharing agreement. Data and documents, including the study protocol, statistical analysis plan, clinical study report, blank or annotated case report forms, will be provided in a secure data sharing environment. For details on submitting a request, see the instructions provided at www.vivli.org**.**

## Abbreviations

ADR: Adverse drug reaction
AE: Adverse events
BP: Blood pressure
CGRP: Calcitonin gene-related peptide
C-SSRS: Columbia-Suicide Severity Rating Scale
CV: Cardiovascular
DCAEs: Discontinuation due to adverse events
EAIR: Exposure adjusted incidence rates
ECG: Electrocardiogram
MedDRA® v.19.1: Medical Dictionary for Regulatory Activities
SAE: Serious adverse events
SMQs: Standard Medical Dictionary for Regulatory Activities queries
TE: Treatment-emergent
TEAEs: Treatment-emergent adverse events
ULN: Upper limit of normal
