# Correction to: Safety and tolerability of monthly galcanezumab injections in patients with migraine: integrated results from migraine clinical studies

**DOI:** 10.1186/s12883-020-01675-7

**Published:** 2020-03-13

**Authors:** Mark E. Bangs, David Kudrow, Shufang Wang, Tina M. Oakes, Gisela M. Terwindt, Delphine Magis, Laura Yunes-Medina, Virginia L. Stauffer

**Affiliations:** 1 Eli Lilly and Company, Indianapolis, IN 46285 USA; 2 California Medical Clinic for Headache, Santa Monica, CA USA; 3 Leiden University Medical Center, Leiden, The Netherlands; 4 Neurology Dept and Pain Clinic (CMTD), CHR East Belgium, Verviers, Belgium

**Correction to: BMC Neurol**

**https://doi.org/10.1186/s12883-020-1609-7**

Following publication of the original article [1], the authors noticed an error in the values for ‘Hypersensitivity SMQ’ and ‘Rash’ in Table 7. The data presented for ‘Hypersensitivity SMQ’ and ‘Rash’ were inadvertently the events that were potentially hypersensitivity rather than the events that were likely hypersensitivity after medical review. The table legend and discussion of the data in the text were and are correct.

**Table 7:** Percentage of patients in integrated double-blind studies who experienced a hypersensitivity event^a,b^

		Galcanezumab	
	Placebo *N* = 1451 n (%)	120 mg *N* = 705 n (%)	240 mg *N* = 730 n (%)
Hypersensitivity SMQ	40 (2.8)	34 (4.8)^c^	32 (4.4)^c^
Rash	15 (1.0)	5 (0.7)	7 (1.0)
Injection site rash	2 (0.1)	6 (0.9)^c^	4 (0.6)
Dermatitis contact	4 (0.3)	3 (0.4)	4 (0.6)
Rhinitis allergic	3 (0.2)	3 (0.4)	4 (0.6)
Hypersensitivity	0 (0.0)	3 (0.4)^c^	2 (0.3)^c^
Injection site hypersensitivity	0 (0.0)	1 (0.1)	3 (0.4)^c^
Dermatitis allergic	0 (0.0)	3 (0.4)^c^	0 (0.0)
Eczema	3 (0.2)	1 (0.1)	2 (0.3)
Rash pruritic	0 (0.0)	2 (0.3)^c^	1 (0.1)
Urticaria	5 (0.3)	2 (0.3)	1 (0.1)
Injection site urticaria	1 (0.1)	1 (0.1)	1 (0.1)
Rash generalized	1 (0.1)	1 (0.1)	1 (0.1)
Rash papulosquamous	0 (0.0)	1 (0.1)	1(0.1)
Dermatitis atopic	1 (0.1)	0 (0.0)	1(0.1)
Eye allergy	0 (0.0)	1 (0.1)	0 (0.0)
Pruritus allergic	0 (0.0)	1 (0.1)	0 (0.0)
Rash erythematous	1 (0.1)	0 (0.0)	1 (0.1)
Rash maculo-papular	1 (0.1)	1 (0.1)	0 (0.0)
Bronchospasm	1 (0.1)	0 (0.0)	0 (0.0)
Conjunctivitis allergic	1 (0.1)	0 (0.0)	0 (0.0)
Drug hypersensitivity	1 (0.1)	0 (0.0)	0 (0.0)

Abbreviations: *MedDRA* Medical Dictionary for Regulatory Activities, *N* number of patients in the analysis population, *n* number of patients within each specific category, *SMQ* standardized MedDRA query

^a^These are likely events per medical review

^b^Categorized by narrow standardized MedDRA®^b^ (v.19.1) query search terms. MedDRA®, the certified Medical Dictionary for Regulatory Activities terminology, is the international medical terminology developed under the auspices of the International Council for Harmonisation of Technical Requirements for Pharmaceuticals for Human Use

^c^Indicates a statistically significant difference (*P* < 0.05) compared with placebo
